# The role of rehabilitation and vitamin D supplementation on motor and psychological outcomes in poststroke patients

**DOI:** 10.1097/MD.0000000000027747

**Published:** 2021-11-12

**Authors:** Michele Torrisi, Lilla Bonanno, Caterina Formica, Francesca Antonia Arcadi, Davide Cardile, Vincenzo Cimino, Placido Bramanti, Elisabetta Morini

**Affiliations:** IRCCS Centro Neurolesi “Bonino Pulejo” – S.S. 113 C.da Casazza, Messina, Italy.

**Keywords:** 25-hydroxyvitamin D, general self-efficacy, neurorehabilitation, poststroke depression, stroke

## Abstract

Post-Stroke depression affects between 12% and 72% of patients who have suffered a stroke. The association between low serum levels of 25-hydroxyvitamin D (25(OH) D) and increased risk of depression is reported in both stroke and non-stroke patients. Similarly, high 25(OH) D levels might be associated with greater functional improvement during rehabilitation program.

We wanted to investigate the effects of an intensive rehabilitation on poststroke outcomes. We wondered if the daily rehabilitation of motor and cognitive functions could also have an effect on mood and functional abilities in addition to or as an alternative to vitamin D supplementation.

We conducted a 12-week, randomized trial, double blind, parallel, monocentric clinical trial of 40 patients undergoing intensive neuro-rehabilitation treatment at a specialized care facility for ischemic or hemorrhagic brain stroke. Participants were randomly assigned, in a 1:1 ratio, to 1 of 2 parallel groups: in the experimental group, 2000 IU/day of oral cholecalciferol was administered; in the control group patients were not taking vitamin D supplementation. Patients underwent a text evaluation to investigate psychological and motor outcomes.

Significant intra-group difference in outcomes measures was found but not between control group and experimental group. In the vitamin D group, we highlighted significant differences between T0 and T1 in calcium (*P* < .001), vitamin D (*P* < .001), in Montgomery Aasberg Depression Rating Scale (*P* = .001), and in Functional Independent Measures (*P* < .001). In the health control group, we found a significant difference in calcium (*P* = .003), vitamin D (*P* < .001), Montgomery Aasberg Depression Rating Scale (*P* = 0.006), in general self-efficacy (*P* = .009), and in Functional Independent Measures (*P* < .001).

Our results show that the beneficial effect on mood and functional recovery is mainly due to neurorehabilitation rather than vitamin D supplementation.

## 1 Introduction

Stroke is one of the most common causes of disability in adults in Western countries,^[1]^ and more than one third of people who survive a stroke will live with severe motor, sensory, and cognitive limitations so as to depend entirely on their family.^[2,3]^ The sudden live changes of stroke survivors causes psychological and behavioral alterations, including depressive mood. Poststroke depression (PSD) affects between 12% and 72% of patients who have suffered a stroke.^[4]^ This variation can be attributed to a number of factors including ethnicity, use of different diagnostic criteria, time interval between the stroke event and assessment, and methodological differences in case selection.^[5]^ PSD can be influenced by several variables, including cognitive level, severity of functional impairment and family support.^[6]^ Some personality features, such as self-efficacy, could play an important role in mediating the degree of PSD.^[7]^ General self-efficacy (GSE) is an overall perception of their ability to deal with adverse events.^[8]^ A significant factor to consider in the pathophysiology of PSD is also the serum level of vitamin D. Vitamin D is a neurosteroid hormone involved in many brain processes including brain development, regulation of neurotrophic factors, neuroplasticity, neuroprotection, and neuroimmunomodulation.^[9–11]^ The association between low serum levels of 25-hydroxyvitamin D (25(OH) D) and increased risk of depression is reported in both stroke and non-stroke patients.^[12–14]^ Similarly, high 25(OH) D levels might be associated with greater functional improvement during rehabilitation program.^[15,16]^ Certainly, PSD and rehabilitation outcomes are strictly correlated. If, on the one hand, the low motivation for treatment, caused by depression, does not allow significant recovery, on the other hand the negative feedback on rehabilitation outcomes could increase the level of PSD. People with PSD have more severe sensorimotor impairments, and poorer abilities to perform essential activities of daily living compared to non-depressed stroke patients.^[17–19]^ This leads to a prolonged period of hospitalization and higher mortality.^[20]^ For these reasons, we believe that it is important to identify the factors related to the PSD and the pharmacological and non-pharmacological strategies to manage it.

In this study, we aimed to investigate the effects of an intensive rehabilitation on poststroke outcomes. Specifically, we have verified if the daily rehabilitation of motor and cognitive functions could also have an effect on mood and functional abilities in addition to or as an alternative to vitamin D supplementation.

## 2 Materials and methods

### 2.1 Participants

**Figure 1:**
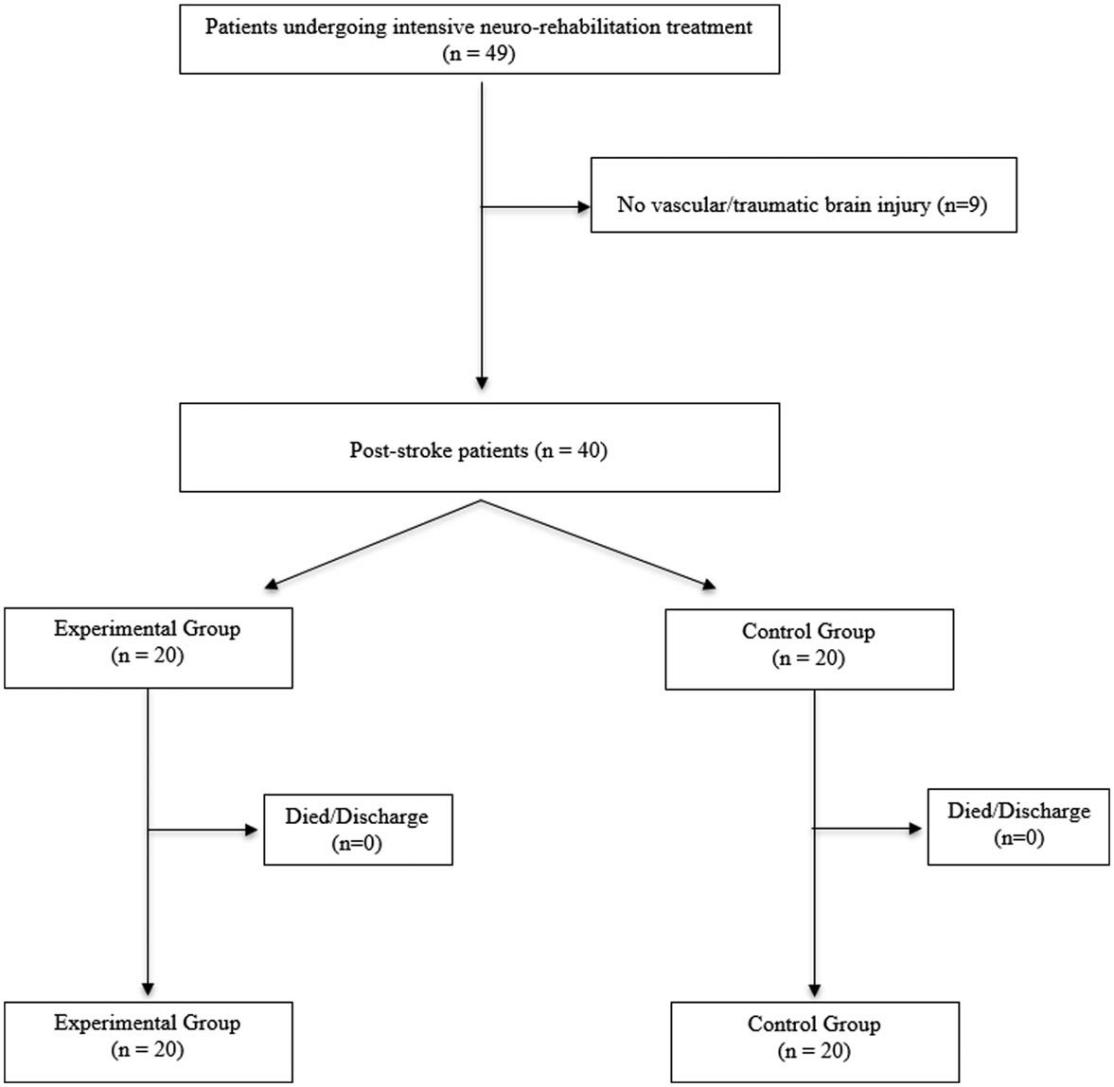
Flow chart of study enrollment.

We conducted a 12-week, randomized trial, double blind, parallel, monocentric clinical trial of 40 patients undergoing intensive neuro-rehabilitation treatment at a specialized care facility for ischemic or hemorrhagic brain stroke (Fig. 1). Participants were randomly assigned, in a 1:1 ratio, to 1 of 2 parallel groups: in the experimental group (EG), 2000 IU/day of oral cholecalciferol was administered; in the control group (CG) patients were not taking vitamin D supplementation. All subjects performed intensive neurorehabilitation. Socio-demographic and characteristics of patients and health CGs are showed in Table 1. The inclusion criteria were: patients with ischemic or hemorrhagic stroke outcomes occurred between 30 and 60 days before and eligible for individual rehabilitation treatment. The exclusion criteria were: Mini Mental State Examination < 15, patients with psychiatric diseases or treated with antidepressants, patients already on vitamin D supplementation, also in combination with calcium, multivitamins or other drugs, and medical conditions that do not allow the neurorehabilitation program. All participants completed the study. The study was approved by Istituito di Ricovero e Cura a Carattere Scientifico (IRCCS) Centro Neurolesi “Bonino-Pulejo” Ethical Committee (approval No. 07/2018) and the informed consent of all patients was obtained.

**Table 1:** Socio-demographic and characteristics clinical of vitamin D and health control group.

		Vitamin D (N = 15)	HC (N = 14)	
		Mean ± SD	Mean ± SD	*P*
Age		59.20 ± 11.38	62.07 ± 10.82	.49
Education		9.07 ± 4.08	11.38 ± 4.59	.18
Gender				
	Male	53.3%	42.9%	
	Female	46.7%	57.1%	
Etiology				
	Ischemic stroke	60%	64.3%	
	Hemorrhagic stroke	40%	35.7%	
Calcium	T0	8.81 ± 0.33	8.69 ± 0.45	.44
	T1	9.13 ± 0.32	9.14 ± 0.22	.93
	*P*	<.001^∗^	.003^∗^	
Vitamin D	T0	19.62 ± 7.92	14.05 ± 4.9	.03^∗^
	T1	30.65 ± 6.36	30.31 ± 4.03	.86
	*P*	<.001^∗^	<.001^∗^	
MADRS	T0	22.13 ± 6.68	21.86 ± 10.43	1
	T1	9.73 ± 4.7	16.36 ± 11.63	.06
	*P*	.001^∗^	.006^∗^	
GSE	T0	28.53 ± 5.59	27.43 ± 7.23	.65
	T1	30.53 ± 3.66	30.5 ± 6.3	.99
	*P*	.16	.009^∗^	
FIM	T0	58.73 ± 32.05	63.45 ± 25.72	.68
	T1	95.53 ± 28.89	80.09 ± 29.12	.19
	*P*	<.001^∗^	<.001^∗^	

FIM = Functional Independent Measures, GSE = general self-efficacy, HC = health control, MADRS = Montgomery Asberg Depression Rating Scale, SD = standard deviation.

∗ *P* < .05.

### 2.2 Procedures

Poststroke patients underwent an intensive rehabilitation training consisting of motor and cognitive training. Specifically, motor-modules included 4 daily rehabilitative sessions for 6 days a week, lasting about an hour. Cognitive rehabilitation included daily sessions based on exercises focused on the enhancement of attention and memory abilities. Psychotherapy and speech therapy was also supplied. Motor rehabilitation was provided both through traditional physiotherapy and through the use of robotic devices such as Lokomat (Hocoma, Volketswil, Switzerland), Armeo Power (Hocoma, Volketswil, Switzerland), or Geo-System (Reha Technology, Olten, Switzerland). The Lokomat is a robotic exoskeleton that consists of a motorized gait insole with computer-controlled linear actuators integrated on each hip and knee joint, a bodyweight support and a treadmill. The guidance can be set by the therapist and determines how much force movements around the knee and hip are forced back towards the reference trajectory. Guidance can be varied between 0% and 100%. The exercises are mainly based on activities in which patients must collect and/or avoid objects randomly distributed in the virtual environment. The G-EO system is a robotic gait training device that allows training to rise and fall. Patients using G-EO are suspended in a harness (removing the weight from the legs) while robotics subjects the legs to the movement of walking. ArmeoSpring exoskeleton (Hocoma AG, Zurich, Switzerland) provides a weight support for the upper extremity and is connected to a Personal Computer with which various virtual-reality based games can be played

### 2.3 Assessment

Patients underwent a text evaluation by the following instruments: GSE scale to assess self-efficacy, Montgomery Aasberg Depression Rating Scale (MADRS) to evaluate mood, and Functional Independent Measures (FIM) to assess the level of disability. This a self-evaluation's scale, the remaining were compiled by the examiner depending on the patient's answers. In addition, serum concentrations of vitamin D and calcium were monitored. All the evaluations were carried out in 2 stages, at the beginning and at the end of the rehabilitation process.

### 2.4 Statistical analysis

Continuous variables were expressed as mean ± standard deviation. Normal data distribution was evaluated using the Shapiro-Wilk normality test. The comparison of clinical variables between the 2 groups was performed with the unpaired Student *t* test or Mann–Whitney *U* test for inter-group analysis. For intra-group analysis, Student paired *t* test or Wilcoxon signed-rank test was used to compare the clinical and neuropsychological variables at baseline and after 3 months in each group (intra-group analysis). Finally, correlation Pearson or Spearman Rank correlation was used for each group in order to assess whether there was a relationship between clinical scores (vitamin D, calcium) and neuropsychological variables (MADRS, GSE, and FIM). Analyses were performed using an open source R3.0 software package (R Foundation for Statistical Computer, Vienna, Austria). A 95% confidence level was set with a 5% alpha error. Statistical significance was set at *P* < .05.

## 3 Results

**Figure 2:**
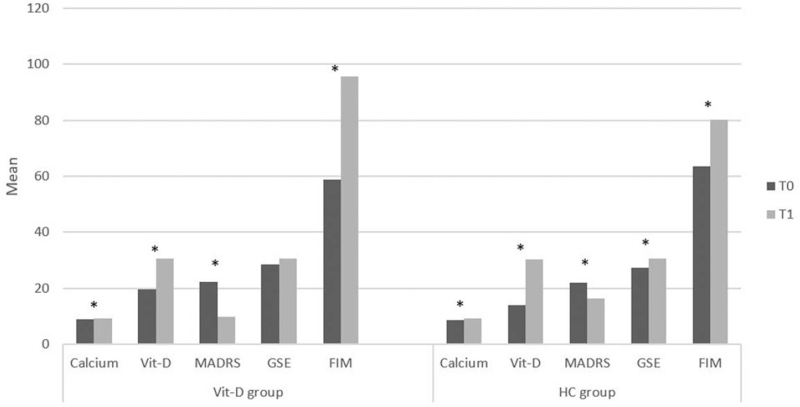
Representation of the clinical and neuropsychological variables in Vitamin D and Health Control (HC) group at T0 (baseline) and T1 (3 months after treatment).

Socio-demographic and characteristics of patients and health CGs are showed in Table 1. Inter-group analysis showed a significant difference in vitamin D (*P* = .03) at T0 and a significant trend in neuropsychological variables as MADRS at T1 (*P* = .06). In the vitamin D group, we highlighted significant differences between T0 and T1 in calcium (*P* < .001), vitamin D (*P* < .001), in MADRS (*P* = .001), and in FIM (*P* < .001) (Fig. 2). In the health control group, we found a significant difference in calcium (*P* = .003), vitamin D (*P* < .001), MADRS (*P* = .006), in GSE (*P* = .009), and in FIM (*P* < .001) (Fig. 2). Pearson correlation showed a negative correlation significant between calcium and MADRS (r = –0.05; *P* = .05) and between vitamin D and MADRS (r = –0.72; *P* = .002) and a positive correlation significant between FIM and vitamin D (r = 0.64; *P* = .01) and a significant trend between vitamin D and GSE (r = 0.48; *P* = .07) and between calcio and FIM (r = 0.49; *P* = .06) in vitamin D group, while, in health control group a negative correlation significant in calcium and MADRS (r = –0.63; *P* = .02) and a positive correlation significant between calcium and GSE (r = 0.56; *P* = .04) were found.

## 4 Discussion

Our study found that patients with stroke who underwent an intensive course of neurorehabilitation showed, at discharge, an improvement in both psychological aspects and functional abilities. This occurred in both patients taking vitamin D supplementation and patients in the CG. With regard to the psychological dimension, we consider these data surprising since the meaningful relationship between vitamin D and depressive disorder is often reported in literature.^[14,21]^ This correlation could be attributable to the presence of numerous receptors for vitamin D in many areas of the human brain, including the cingulate cortex, hippocampus, hypothalamus, and substantia nigra, which have been involved in the pathophysiology of depression.^[22,23]^ Some authors have hypothesized that vitamin D has a regulatory action on serotonin concentrations, a tryptophan derived neurotransmitter involved in mood and behavior.^[24]^ Other findings show that vitamin D can be considered an epigenetic factor in the production and modulation of oxytocin,^[25]^ a hormone considered involved in mood disorders, when its concentrations are insufficient.^[26]^ Besides, low serum concentrations of 25(OH) D are very common in depressed population^[14]^ and this relationship has also been investigated with regard to other psychological or psychiatric disorders such as schizophrenia,^[27]^ autism spectrum disorders,^[28]^ substance dependence,^[29]^ sleep disorders,^[30]^ and anxiety.^[31]^ Concerning this, several meta-analysis and systematic reviews have evaluated the impact of vitamin D supplementation, mainly in the form of cholecalciferol (vitamin D3), for the treatment of depressive symptoms. Nevertheless, the results are not consistent.^[32–34]^ The discrepancy of results may depend on several factors such as: sample size, diagnostic tools used, and method of supplementation (oral or parenteral), average 25(OH) D serum level at baseline, and co-occurrence of other clinical diseases such as immune, cardiovascular, and neurological. With regard to neurologic comorbidity, few studies have focused on the relationship between serum vitamin D levels and the development of PSD, noting that stroke patients who reported 25[OH] D deficiency (<20 ng/mL) or insufficiency (20–30 ng/mL) within 24 hours of entering a Stroke Unit were more likely to report PSD at 1 month^[35]^ or 6 months^[12]^ after the acute event. These studies, however, did not specify whether these patients had been studied during a rehabilitation course. To the best of our knowledge, this is the first report exploring the possible association between serum vitamin D levels and the development of PSD among patients underwent a daily intensive neurorehabilitation treatment. Previously, the impact of vitamin D levels on poststroke rehabilitation has only been investigated on functional abilities,^[16,36,37]^ leaving out psychological repercussions. The improvement in mood that we detected at discharge in patients in both experimental and CGs suggest that this change was due to intensive rehabilitation rather than vitamin D supplementation. This finding, as well as unexpected, also makes an original contribution to the literature on this relationship since previous studies have usually observed the influence of mood on functional recovery^[17,38]^ and not vice versa. However, it should be pointed out that patients, who have taken vitamin D supplementation, show a more marked variation, as shown in Table 1. With regard to the functional aspect, our data also confirm a greater influence of rehabilitation than Vit D supplementation, as the increase in FIM scores occurs in both groups although it is more accentuated in the experimental. With regard to perceived GSE, we found a significant improvement between T0 and T1 only in the CG. This means that, in this case, rehabilitation did not exert any influence, probably because GSE is a construct related to personality and therefore is much more stable than mood, which, instead, can be much more variable and reactive to contingent conditions. Moreover, there is no correlation between vitamin D supplementation and GSE in the EG. On the other hand, the significant increase in plasma calcium levels and in FIM scores, observed in both groups, may also be explained by intensive rehabilitation. In addition, we found that increased serum calcium levels correlated with improved mood. This finding has no previous evidence in the literature, although it is likely to be related to the close connection of calcium with vitamin D metabolism,^[39]^ which was increased in both groups studied. In conclusion, our results show that the beneficial effect on mood and functional recovery is mainly due to neurorehabilitation rather than vitamin D supplementation. However, considering that patients in the EG show a more evident improvement, it cannot be said that vitamin D supplementation is very irrelevant. It is also fair to point out that several studies^[40–44]^ have shown that low plasma levels of Vit D are associated with an increased risk of stroke. Thus, vitamin D supplementation can still be considered for stroke prevention in predisposed individuals.

In conclusion, our findings suggest the importance of poststroke neurorehabilitation, not only for motor recovery. For this reason, it is important that patients be transferred to a rehabilitation center as soon as possible after treatment of the acute phase. This also allows avoiding a chronicity of the depressive state and the associated effects on the general well-being of the patient.

## Author contributions

**Conceptualization:** Lilla Bonanno, Elisabetta Morini.

**Data curation:** Michele Torrisi, Lilla Bonanno, Caterina Formica, Francesca Antonia Arcadi, Davide Cardile, Vincenzo Cimino.

**Formal analysis:** Lilla Bonanno.

**Investigation:** Caterina Formica.

**Methodology:** Michele Torrisi, Elisabetta Morini.

**Resources:** Davide Cardile.

**Supervision:** Lilla Bonanno, Francesca Antonia Arcadi, Placido Bramanti.

**Validation:** Caterina Formica, Placido Bramanti, Elisabetta Morini.

**Visualization:** Placido Bramanti, Elisabetta Morini.

**Writing – original draft:** Michele Torrisi, Lilla Bonanno, Elisabetta Morini.

**Writing – review & editing:** Vincenzo Cimino, Placido Bramanti.
